# What matters in laparoscopic hepatectomy for lesions located in posterosuperior segments? Initial experiences and analysis of risk factors for postoperative complications: a retrospective cohort study

**DOI:** 10.1007/s00464-025-11674-9

**Published:** 2025-04-30

**Authors:** Patrick Téoule, Niccolo Dunker, Vanessa Gölz, Erik Rasbach, Christoph Reissfelder, Emrullah Birgin, Nuh N. Rahbari

**Affiliations:** 1 Department of Surgery, Universitätsmedizin Mannheim, Medical Faculty Mannheim, Heidelberg University, Theodor-Kutzer-Ufer 1-3, 68167 Mannheim, Germany; 2 DKFZ-Hector Cancer Institute at University Medical Center Mannheim, Mannheim, Germany; 3 Present Address: Department of General and Visceral Surgery, Ulm University Hospital, Ulm, Germany

**Keywords:** Clavien–Dindo, Laparoscopic liver resection, Intraoperative transfusion perioperative outcome

## Abstract

**Background:**

Laparoscopic liver resection (LLR) for lesions in the posterosuperior segments (PSS) is challenging. Identifying and minimizing risk factors for postoperative morbidity and mortality is crucial. This retrospective cohort study shares initial experiences with LLR of the PSS (VII, VIII, IVa) and wants to identify risk factors for clinically relevant postoperative complications (Clavien–Dindo grade ≥ III) in these patients.

**Methods:**

We reviewed our prospective database for all patients who underwent LLR with at least one lesion in the PSS (April 2018–October 2022). Uni- and multivariate analyses were carried out using binary logistic regression analysis.

**Results:**

110 patients underwent LLR of the PSS. Median age was 67 years (IQR 59–76); 62% were male (*n* = 68), with a median BMI of 26 (IQR 23–30). The most frequent indications for LLR were primary liver cancer (37%) and colorectal liver metastasis (36%). Median operating time was 211 min (IQR 135–281) with a median blood loss of 460 mL (IQR 240–1200). Postoperative length of stay was 6 days (IQR 4–8). Clinically relevant postoperative complications were present in 20 patients (18%) with a 90-day mortality rate of 5% (*n* = 6). Multivariate analyses identified ASA ≥ III (OR 3.23 [95%CI 1.03–10.09]; *p* = 0.043), diabetes (OR 4.31 [95%CI 1.20–15.49]; *p* = 0.025), and intraoperative transfusion of packed red blood cells (PRBC) (OR 4.80 [95%CI 1.01–22.86]; *p* = 0.049) as risk factors for Clavien–Dindo grade ≥ III complications.

**Conclusion:**

ASA ≥ III status, diabetes, and intraoperative PRBC transfusion are associated with an increased risk of Clavien–Dindo grade ≥ III complications in patients undergoing LLR in PSS. Preoperative optimization should include diabetes management, screening for anemia with appropriate supplementation, and comprehensive risk counseling for ASA ≥ III patients. Additionally, minimizing intraoperative PRBC transfusion should remain a key perioperative goal.

Laparoscopic hepatectomy has gained increasing popularity among hepatobiliary surgeons due to lower morbidity rates as compared to open hepatectomy. However, laparoscopic hepatectomy of lesions located in the posterosuperior segments (PSS), i.e., VII, VIII and IVa, are still considered as challenging resections owing to the close proximity to hepatic veins with high risk of major bleeding, limited visualization, and working space [1, 2]. Therefore, the international Southampton consensus guidelines and the literature recommend that hepatectomy of posterosuperior segments be reserved for experts in minimally invasive liver surgery [3–5]. Some studies previously addressed the safety and feasibility of laparoscopic liver resection (LLR) in the PSS, resulting in improved short-term outcomes by choosing the laparoscopic approach [6–10]. Although patients undergoing LLR of PSS had fewer clinically relevant complications [6, 7], a shorter length of hospital stay compared to the open approach [6, 7, 9, 10], and significantly less intraoperative blood loss [6, 8, 10], concerns about LLR in the PSS have not been entirely eliminated. The interventions’ complexity can lead to adverse events during surgery, with a negative effect on the acceptance of the approach. The identification of potentially modifiable risk factors may help to minimize complications and to improve patient outcomes.

To our knowledge, this is the first single-center study in a western population investigating potential risk factors associated with postoperative complications, in patients undergoing LLR for lesions located in the PSS. Therefore, it was the aim of this study to identify potential risk factors associated with clinically relevant postoperative complications (Clavien–Dindo grade ≥ III) in patients undergoing laparoscopic hepatic resection in the PSS. Furthermore, we aim to share our experience with LLR of the PSS at a European tertiary center.

## Materials and methods

### Study design

The study has been retrospectively registered within the German Clinical Trials Register (DRKS00032298). The ethical review committee of the Heidelberg University, Medical Faculty Mannheim, approved this retrospective cohort study of a prospectively recorded data base (2019-753N-MA) and it has been carried out in accordance with the Declaration of Helsinki. Furthermore, the study was conducted in line with the STROCSS guidelines [11].

Informed consent for surgery as well as for data collection and analysis was given by all patients. All consecutive patients who underwent LLR between April 2018 and October 2022 at the Department of Surgery, University Hospital Mannheim, Heidelberg University, Medical Faculty Mannheim were screened. Patients with at least one resected lesion in the PSS (VII, VIII, and IVa) were included in this study [12]. Exclusion criteria were laparoscopic right or left hepatectomy and patients undergoing laparoscopic two-staged hepatectomy.

### Preoperative assessment, definitions, and outcomes

Demographic and clinical characteristics included age, sex, BMI, and preoperative status of patients according to the ASA status classification. Other factors were diabetes mellitus, cardiovascular comorbidities (hypertension, atrial fibrillation, coronary heart disease, heart failure, valve diseases), pulmonary comorbidities (chronic obstructive pulmonary disease, asthma, pulmonary fibrosis, hypertension), renal insufficiency, neurological/psychological comorbidities (history of stroke, depression), and smoking status. Medication use (antihypertensives, platelet inhibitors, diabetes medication, lipid-lowering agents, endocrinological medication [thyroids, hormones], proton pump inhibitors, bronchodilators, neurological/psychiatric medication, steroids) was also recorded. Additionally, the indication for surgery (benign vs. malignant) as well as history of previous abdominal surgery and/or hepatic resection were noted [13].

Preoperative laboratory tests assessed included (reference values in parentheses) albumin [≥ 35 g/L], bilirubin [$\le$ 1.2 mg/dL], alanine aminotransferase [0–50 U/L), aspartate aminotransferase [0–50 U/L), creatinine [$\le$ 1.4 mg/dL], hemoglobin [≥ 13 g/dL], platelets [145–348 × 10^9^/L], and international normalized ratio (INR) [0.9–1.15]. A multidisciplinary board confirmed the indication for surgical resection in all oncological patients. In case of hepatocellular carcinoma (HCC), the etiology, e.g., alcohol, non-alcoholic steatohepatitis, autoimmune, and viral, was documented. The revised Child–Pugh scoring system was used in the case of cirrhosis [14].

Endpoints included operating time, total blood loss, extent of resection, intra- and postoperative transfusion (within 48 h after surgery), postoperative morbidity and mortality, pathologic characteristics as well as postoperative length of hospital stay. The extent of liver resection was classified using the Brisbane nomenclature and Couinaud’s segmentation. Anatomic liver resections were performed according to Couinaud’s portal segmentation corresponding to an entirely removal of a portal territory with its respective parenchyma [15]. The decision between anatomic and non-anatomic hepatectomy was based on the tumor related factors (infiltration of primary or secondary portal/biliary branches), future liver remnant, and surgeon’s discretion. Major hepatectomy was defined as anatomic resection of at least three liver segments [16]. The IWATE score was used to evaluate the difficulty of LLR in the PSS [17].

Postoperative complications within 90 days were graded using the Clavien–Dindo classification [18]. Clinically relevant postoperative complications were defined as Clavien–Dindo grade ≥ III. Hepatectomy-specific complications, such as post-hepatectomy hemorrhage (PHH), post-hepatectomy bile leakage (PHBL), and post-hepatectomy liver failure (PHLF), were assessed according to the criteria of the International Study Group of Liver Surgery (ISGLS) [19–21]. The readmission rate was defined as hospital readmission within 90 days after the index operation.

### Surgical and anesthesiologic management

**Fig. 1:**
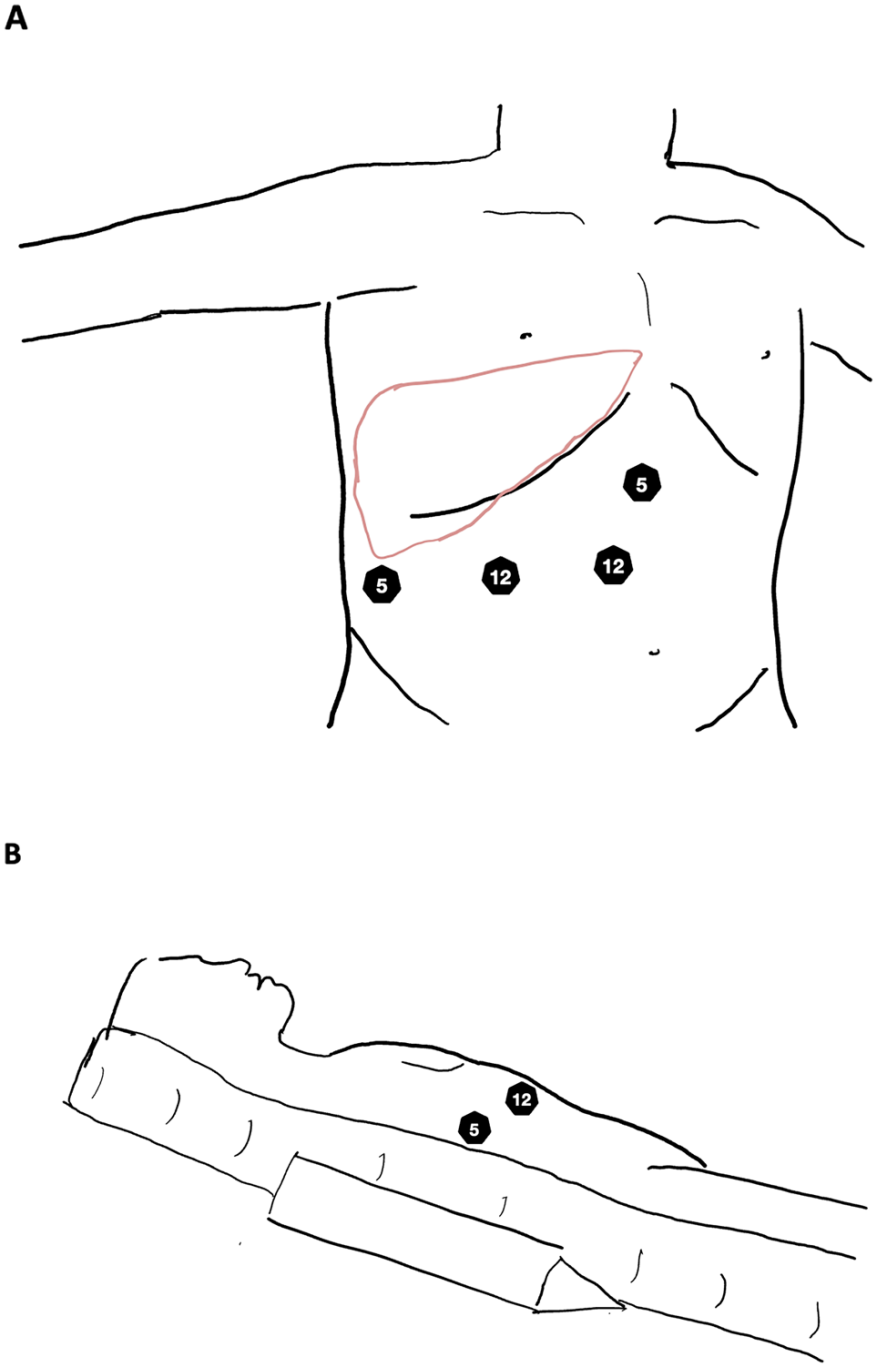
Trocar placement (**A**) and patient position (**B**). Patients were positioned in the French position with reversed Trendelenburg, slightly tilted left, and secured on a vacuum mattress. A wedge cushion ensured optimal exposure of the right hemithorax and abdomen. Typically, four trocars (two 12 mm and two 5 mm) were arranged in a reversed L-shape, which might be used for laparotomy. For clarity, the illustration of the laterally displaced arm has been omitted in **B**

Surgery was performed under general anesthesia. Patients were positioned in the French position with reversed Trendelenburg, slightly tilted left, and secured on a vacuum mattress. A wedge cushion ensured optimal exposure of the right hemithorax and abdomen. The right arm was carefully placed laterally, maintaining a safe 90-degree range (Fig. 1). Generally, four abdominal trocars were used (two 12 mm and two 5 mm ports), with additional trocars placed at the surgeon’s discretion if needed. No trans-thoracic or intercostal ports were used. A capnoperitoneum of 12 mmHg was established, and the abdomen was explored for extrahepatic disease. Intraoperative ultrasound was routinely performed to detect previously unrecognized lesions and to delineate the resection planes. For exposure of the PSS the right hemiliver was completely mobilized.

Liver transection was carried out under low central venous pressure [22–26], using the crush-clamp technique in combination with an energy device [24], as described previously. Vascular and biliary structures larger than 2 mm in diameter were divided using titanium (®Braun) or Hem-o-Lok (®WECK) clips. The specimen was retrieved through a Pfannenstiel incision, using a tissue bag. No abdominal drains were placed. Patients who underwent major resections were monitored on an intermediate care unit overnight. Oral fluid intake was allowed on the day of surgery, with food intake resuming on the first postoperative day.

### Statistical analysis

Categorical data were presented by absolute and relative frequencies (percentage) and compared using Pearson’s *χ*^2^ test or Fisher’s exact test. Quantitative data were summarized as mean (standard deviation) or median (interquartile range (IQR) or 95% confidence interval (95%CI) and compared using the unpaired 2-tailed *t* test or Mann–Whitney *U* test, based on the distribution pattern. For univariate comparisons, *χ*^2^ analysis or Fisher’s exact tests were used to evaluate categorical variables; alternatively, continuous variables were analyzed using the Student’s *t* test and Wilcoxon rank-sum test for normally and non-normally distributed data, respectively.

Multivariate binary logistic regression analysis was performed to identify risk factors associated with clinically relevant postoperative complications (Clavien–Dindo ≥ III) (*p* < 0.05 for entry; *p* > 0.05 for removal). To assess the independence of risk factors, significant pre- and intraoperative variables from the univariate analysis were included in the multivariate analysis. Operative time and intraoperative blood loss were excluded from both uni- and multivariate analyses due to their strong dependence on patient and tumor characteristics, as well as the type of resection. Their inclusion could introduce bias, as prolonged operative time and high blood loss often reflect more technically challenging resections rather than serving as independent predictors of complications. Additionally, operative time is influenced by external factors, such as operating room logistics. Instead of intraoperative blood loss, we included transfusions in the uni- and multivariate analyses, as postoperative transfusions have a greater impact on patient outcomes. *P* values < 0.05 were defined as statistically significant. SAS-Version 9.4 (SAS Institute) was used for all statistical analyses.

## Results

### Patient characteristics

**Fig. 2:**
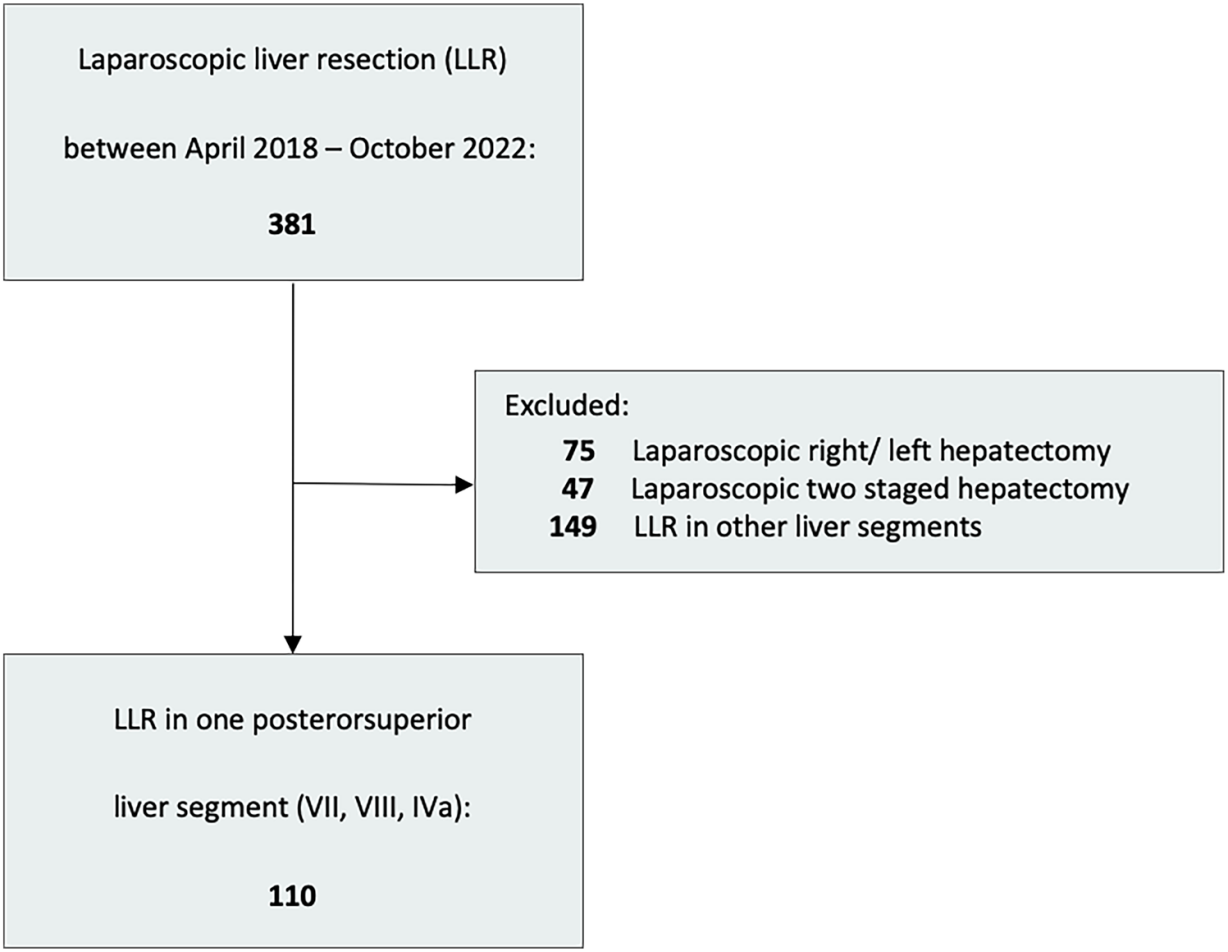
Patient flow chart

Out of 381 patients who underwent laparoscopic hepatectomy, a total of 110 patients underwent LLR for lesions in the PSS and were included in this study (Fig. 2). Patient characteristics are outlined in Table 1. The median age was 67 years (IQR 59–76 years), with a majority being male (*n* = 68, 62%). Cardiovascular comorbidities were present in 66 patients (60%), diabetes in 28 patients (25%), and pulmonary comorbidities in 17 patients (15%). The most common medications were antihypertensives (*n* = 62; 65%), followed by platelet inhibitors (*n* = 38; 35%).

**Table 1:** Patient characteristics

	N = 110
Age (years)	67 (59–76)
Sex ratio (male:female)	68:42
BMI (kg/m^2^)	26 (23–30)
ASA $\ge$ III	50 (45)
Cardiovascular comorbidities	66 (60)
Diabetes	28 (25)
Pulmonary comorbidities	17 (15)
Neurological/psychiatrical comorbidities	9 (8)
Renal insufficiency	4 (4)
Smoking	16 (15)
Cirrhosis	24 (22)
Child A	21 (19)
Child B	3 (3)
Antihypertensive medication	62 (65)
Platelet inhibitors	38 (35)
Diabetes medication	28 (25)
Lipid lowering medication	25 (23)
Proton pump inhibitors	24 (22)
Bronchodilative medication	7 (6)
Neurological/ psychiatrical medication	9 (8)
Steroid medication	5 (5)
Diagnosis	
Hepatocellular carcinoma	30 (27)
Intrahepatic cholangiocarcinoma	11 (10)
Colorectal liver metastasis	40 (36)
Other	17 (15)
Benign	12 (11)
Largest tumor size (cm)	2.8 (1.8–4.4)
Previous abdominal surgery	75 (68)
Previous hepatic resection	26 (24)
Preoperative blood values	
Albumin (g/L)	37.0 (34.0–40.3)
Bilirubin (mg/dL)	0.5 (0.4–0.7)
Gamma-glutamyl transferase (U/L)	49 (28–107)
Alanine aminotransferase (U/L)	29 (20–43)
Aspartate aminotransferase (U/L)	26 (20–35)
International normalized ratio	1.0 (1.0–1.1)
Creatinine (mg/dL)	0.8 (0.7–1.0)
Hemoglobin (g/dL)	13.6 (11.4–14.5)
Platelets (10E9/L)	220 (172–291)
Difficulty score^a^	7 (5–10)
Difficulty level^a^	
Intermediate	54 (49)
Advanced	27 (25)
Expert	29 (26)

Data are shown as *n* (%) or median (interquartile range)

*ASA* American Society of Anesthesiologists, *BMI* body mass index, *kg* kilogram, *m* meter, *Other* other secondary liver malignancies: six breast cancer, two pancreatic adenocarcinoma, two leiomyosarcoma of the uterus and, respectively, one each: laryngeal carcinoma, adenocarcinoma of the gastroesophageal junction, distal cholangiocarcinoma, clear renal cell carcinoma, prostate cancer, neuroendocrinal tumor, and forehead squamous cell carcinoma, *Benign* benign lesions: six echinococcus cyst, three hemangiomas, two focal nodal hyperplasia, and one primary sclerosing cholangitis, *cm* centimeter, *g* gram,, *L* liter, *mg* milligram, *dL* deciliter, *U* units

^a^IWATE criteria

Primary liver malignancy was present in a total of 41 patients (37%) and secondary liver malignancy in 57 patients (52%). In case of primary liver malignancy, 30 patients had HCC and eleven had intrahepatic cholangiocarcinoma, whereas CRLM were the most common indication for surgery in case of secondary liver malignancy. A total of 24 patients (22%) had liver cirrhosis, predominantly Child A 88% (*n* = 21) and three patients (12%) Child B. Alcohol and viral hepatitis each caused HCC in twelve patients (11%), while steatohepatitis was present in a total of six patients (5%). The majority of patients (*n* = 75, 68%) had previous abdominal surgeries, including 26 (24%) with previous hepatectomies. The median difficulty score was 7 (IQR 5–10), with 51% (*n* = 56) classified as advanced/ expert LLR cases.

### Operative procedures and intraoperative outcomes

The operative details are summarized in Table 2. Major hepatectomy was performed in twelve patients (11%). A slightly higher proportion of patients (*n* = 57, 62%) underwent an anatomic resection. Among the 110 patients, eight (7%) underwent an extrahepatic resection, including four (4%) partial diaphragm resections, four (4%) colonic, one (1%) pancreas, and one (1%) kidney/adrenal gland resection. The combined colonic resections were planned due to subtotal stenotic primary tumors.

**Table 2:** Operative characteristics

	*N* = 110
Surgical procedure	
Anatomic resections	57 (52)
Number of anatomic resections	2 (1–2)
Segmentectomy	25 (23)
Bisegementectomy	20 (18)
Right anterior sectionectomy	4 (4)
Right posterior sectionectomy	11 (10)
Other bisegmentectomy	5 (5)
Other segmentectomy of 3 segments	8 (7)
Central hepatectomy	4 (4)
Non-anatomic resections	53 (48)
Number of non-anatomic resections	1 (1–2)
One partial resection	27 (25)
Two or more partial resection	26 (24)
Operating time (min)	211 (135–281)
Blood loss (mL)	460 (240–1200)
Pringle maneuver	64 (58)
Duration, min	57 (30–87)
IVC-clamping	10 (9)
Duration, min	64 (59–68)
Intraoperative PRBC transfusion	30 (27)
Intraoperative FFP transfusion	41 (37)
R1 status^a^	5 (5)

Data are shown as *n* (%) or median (interquartile range)

*min* minutes, *mL* milliliter, *PRBC* packed red blood cells, *FFP* fresh frozen plasma, *IVC* infrahepatic vena cava clamping

^a^R1-status of all resections due to malignancy

Median blood loss was 460 mL (IQR 240–1200 mL) and median operating time 211 min (IQR 135–281 min). Intraoperative transfusion of packed red blood cells or fresh frozen plasma was median 0 (IQR 0–1) each with 30 (27%) and 41 (37%) patients, respectively, receiving transfusions. The median age of the 30 patients who received an intraoperative transfusion of packed red blood cells was 68 years (IQR 63–79 years), and 80% (*n* = 24) had preoperative anemia. Nearly three-quarters of these patients (*n* = 22, 73%) had at least one of the following comorbidities: cardiovascular disease, pulmonary disease, or diabetes. Positive resection margins were observed in five patients (5%), involving one case each of intrahepatic cholangiocarcinoma and HCC and three cases of colorectal liver metastases, all parenchymatous.

Conversion to open surgery was required in eight cases (7%). Five out of the eight patients with the need of laparotomy had Child B liver cirrhosis with severe rigid livers, intrahepatic shunts, and consecutive diffuse intraoperative hemorrhage. All of these five patients received intraoperative transfusion of packed red blood cells. Two patients underwent conversion to laparotomy at the beginning of surgery due to extensive adhesions with the need of adhesiolysis of several hours. One patient required partial vena cava resection and reconstruction due to tumor infiltration.

### Postoperative outcome

Postoperative outcomes are summarized in Table 3. The majority of patients (*n* = 58, 53%) underwent LLR in the PSS without any complication according to the Clavien–Dindo classification. Clinically relevant postoperative complications (Clavien–Dindo ≥ III) were present in 20 patients (18%). Hepatectomy-specific complications included PHBL (ISGLS) Grade B/C in nine out of ten patients, PHLF (ISGLS) Grade B/C in seven out of nine, and PPH (ISGLS) Grade B/C in three out of six. Invasive re-interventions, i.e., radiologic drainage were required in 18 patients and relaparotomy in eight.

**Table 3:** Postoperative outcome

	*N* = 110
Postoperative complications^a^	
None	58 (53)
Grade I	20 (18)
Grade II	12 (11)
Grade IIIA	5 (5)
Grade IIIB	5 (5)
Grade IV	4 (4)
Grade V	6 (5)
Clinically relevant complications^a^	
$<$ Grade III	32 (29)
$\ge$ Grade III	20 (18)
Type of complications	
Posthepatectomy bile leakage^b^	10 (9)
Posthepatectomy liver failure^b^	9 (8)
Posthepatectomy hemorrhage^b^	6 (5)
Intraabdominal abscess	8 (7)
Wound infection	6 (5)
Invasive interventions	18 (16)
Radiologic drainage	13 (12)
Endoscopic intervention	8 (7)
PTCD	1 (1)
Postoperative PRBC transfusion^c^	5 (5)
Postoperative FFP transfusion^c^	6 (5)
Relaparotomy	8 (7)
Readmission	8 (7)
IMC stay (days)	1 (1–2)
Postoperative length of stay (days)	6 (4–8)

Data are shown as *n* (%) or median (interquartile range)

^a^Clavien–Dindo classification

^b^International Study Group of Liver Surgery; *PTCD* percutaneous transhepatic cholangial drainage, *PRBC* packed red blood cells, *FFP* fresh frozen plasma

^c^Within < 48 h after surgery; *IMC* Intermediate Care Unit

The 90-day mortality rate was 5% (*n* = 6), all due to sepsis with multiorgan failure. These patients had extensive comorbidities, including cardiorenal dysfunction and insulin-dependent diabetes mellitus. The median age of the six multimorbid patients was 72 years (IQR 63–82). The median POD to death was 41 (IQR 19–64). Four of these cases followed anatomic LLR in the PSS, while two followed non-anatomic resections. The first patient who died from sepsis and multiorgan failure after an anatomic hepatectomy in the PSS did not recover from PHH Grade C and developed a superinfected intraabdominal hematoma. The second patient developed pneumonia, worsening Child A liver cirrhosis, and a Grade C PHBL. The third and fourth patients within the anatomic LLR in the PSS did not recover from Grade C PHBL and required relaparotomy due to colonic ischemia. The first patient who died due to sepsis with multiorgan failure after non-anatomical LLR in the PSS resection did not recover from Grade C PHBL and multiple progressive cholangiocellular abscesses. The second patient also did not recover from Grade C PHH following a converted atypical hepatectomy and required relaparotomy due to a superinfected hematoma. Postoperative transfusion of packed red blood cells and fresh frozen plasma received six patients (5%) each.

The median postoperative length of hospital stay was 6 days (IQR 4–8 days). The median length of stay on the intermediate care unit was one day (IQR 1–2 days). Overall, ten (9%) patients were administered to an intensive care unit (ICU), with a median stay of two days.

### Predictors of postoperative outcome

To evaluate the independent predictive value of clinicopathological factors associated with the occurrence of clinically relevant postoperative complications (Clavien–Dindo ≥ III) for LLR of the PSS, we performed both univariate and multivariate analyses (Table 4).

**Table 4:** Clinicopathological factors associated with clinically relevant complications (Clavien–Dindo ≥ III)

	Univariate			Multivariate	
	Clavien–Dindo ≥ III (*N* = 20)	vs. Clavien–Dindo ≤ 2 (*N* = 90)	*P* value	OR (95% CI)	*P* value
Age (years)	71 (63.0–78.0)	66 (58.0–75.0)	0.251		
Male sex	15 (75)	53 (58)	0.180		
BMI > 30 (kg/m^2^)	6 (30)	21 (23)	0.449		
ASA ≥ III	16 (80)	34 (38)	**< 0.001**	3.239 (1.039–10.099)	**0.043**
Cardiovascular comorbidities	14 (70)	52 (58)	0.428		
Diabetes	11 (55)	17 (19)	**< 0.001**	4.317 (1.203–15.495)	**0.025**
Pulmonary comorbidities	3 (15)	14 (16)	0.652		
Neurological/psychiatrical comorbidities	1 (5)	8 (9)	0.543		
Renal insufficiency	1 (5)	3 (3)	0.749		
Smoking	4 (20)	12 (13)	0.444		
Cirrhosis	7 (35)	17 (19)	0.115		
Antihypertensive medication	13 (65)	39 (43)	0.497		
Platelet inhibitors	8 (40)	30 (33)	0.686		
Lipid lowering medication	4 (20)	21 (23)	0.642		
Proton pump inhibitors	4 (20)	20 (22)	0.868		
Bronchodilative medication	0	7 (8)	0.180		
Neurological/psychiatrical medication	1 (5)	8 (9)	0.534		
Primary liver malignancy	11 (55)	30 (33)	0.070		
Secondary liver malignancy	9 (45)	48 (53)	0.500		
Largest tumor size (cm)	3.5 (2.0–6.5)	2.7 (1.8–4.2)	0.250		
Difficulty score^a^	7 (5–9)	7 (5–10)	0.627		
Previous abdominal surgery	12 (60)	66 (84)	0.385		
Previous hepatic resection	6 (30)	20 (22)	0.459		
Preoperative blood values					
Albumin (g/L)	36.5 (31.6–38.2)	37.3 (34.1–40.6)	0.078		
Bilirubin (mg/dL)	0.5 (0.4–0.9)	0.5 (0.4–0.7)	0.585		
Gamma-glutamyl transferase (U/L)	70 (37–134)	46 (27–99)	0.228		
Alanine aminotransferase (U/L)	36 (20–50)	26 (20–39)	**0.023**	1.005 (0.995–1.016)	0.296
Aspartate aminotransferase (U/L)	48 (24–60)	48 (19–38)	0.065		
International normalized ratio	1.0 (1.0–1.1)	1.1 (1.0–1.1)	0.792		
Creatinine (mg/dL)	0.9 (0.7–1.0)	0.8 (0.7–1.0)	0.569		
Hemoglobin (g/dL)	13.3 (10.1–14.2)	13.6 (12.0–14.6)	0.156		
Platelets (10E9/L)	207 (143–309)	224 (176–283)	0.483		
Anatomic LLR vs. non-anatomic LLR	10 (50)	47 (52)	0.857		
Segmentectomy vs. bisegmentectomy or more	5 (25) vs. 5 (25)	20 (22) vs. 27 (30)	0.667		
One vs. two or more partial resections	4 (20) vs. 6 (30)	23 (25) vs. 20 (22)	0.442		
Major hepatectomy^b^	3 (15)	9 (10)	0.516		
Extrahepatic resection	3 (15)	5 (6)	0.141		
Pringle maneuver	11 (55)	53 (59)	0.750		
Duration, min	75 (40–100)	45 (28–82)	0.256		
IVC-Clamping	2 (10)	8 (9)	0.876		
Duration, min	107 (83–130)	67 (42–96)	0.667		
Intraoperative PRBC transfusion	12 (60)	18 (20)	**< 0.001**	4.805 (1.010–22.868)	**0.049**
Intraoperative FFP transfusion	13 (65)	28 (31)	**0.005**	0.791 (0.160–3.935)	0.775
Postoperative PRBC transfusion^c^	2 (10)	3 (3)	0.195		
Postoperative FFP transfusion^c^	2 (10)	3 (3)	0.195		
Conversion to open surgery	3 (15)	5 (6)	0.141		

Data are shown as *n* (%) or median (interquartile range)

*LLR* laparoscopic liver resection, *OR* odds ratio, *CI* confidence interval, *ASA* American Society of Anesthesiologists *BMI* body mass index, *kg* kilogram, *m* meter, *cm* centimeter

^a^IWATE Criteria; *g* gram, *L* liter, *mg* milligram, *dL* deciliter, *U* units

^b^Defined as resection of more than two anatomic segments; *IVC* infrahepatic vena cava clamping, *min* minutes, *PRBC* packed red blood cells, *FFP* fresh frozen plasma

^c^Within < 48 h after surgery; Values in bold, *p* < 0.05

Univariate analysis identified ASA grade $\ge$ III (*p* < 0.001), diabetes (*p* < 0.001), preoperative alanine aminotransferase value (*p* = 0.023), and intraoperative transfusion of packed red blood cells (*p* < 0.001) as well as fresh frozen plasma (*p* = 0.005) to be significantly associated with clinically relevant postoperative complications (Clavien–Dindo ≥ III) (all *p* < 0.05).

Multivariate analyses identified ASA grade $\ge$ III (odds ratio (OR) 3.23 [95% CI 1.03–10.09]; *p* = 0.043), diabetes (OR 4.31 [95% CI 1.20–15.49]; *p* = 0.025) and intraoperative transfusion of packed red blood cells (OR 4.80 [95% CI 1.01–22.86]; *p* = 0.049) as independent factors for Clavien–Dindo ≥ III complications.

## Discussion

In this retrospective cohort study, we evaluated the impact of potential risk factors associated with the occurrence of clinically relevant postoperative complications (Clavien–Dindo grade ≥ III) following laparoscopic hepatectomy for lesions located in the PSS. Moreover, we aim to share our experiences with LLR of the PSS at a European tertiary center.

We conducted multivariate analyses to identify risk factors for Clavien–Dindo ≥ III complications. The presence of diabetes, ASA grade $\ge$ III, and intraoperative transfusion of packed red blood cells were independent predictors of clinically relevant postoperative complications following LLR of the PSS. These findings are in line with previous studies focusing on predictors of complications, particularly in open hepatectomy and LLR of other segments. Prior research has indicated higher rates of PHLF in patients with type 2 diabetes and increased major complication rates in those with insulin-dependent diabetes mellitus [27, 28]. In our study, 39% of patients (eleven out of 28) had insulin-dependentdiabetes mellitus. Out of the nine patients with clinically relevant postoperative complications and concomitant diabetes, six (66%) patients were insulin dependent.

The ASA score is a widely used measure of patient’s overall physical health. Furthermore, it is significantly and independently associated with medical complications and mortality following various surgical procedures [29]. In accordance with this, an ASA score ≥ III is a well-known predictor of postoperative morbidity after hepatectomy and was confirmed by our study [30–32].

Intraoperative transfusion of PRBCs is a recognized risk factor for postoperative morbidity after liver resection, as demonstrated in various studies and meta-analyses across different surgical approaches in liver surgery [33, 34]. This study, through multivariate analyses, is to our knowledge the first one to reveal the negative impact of intraoperative PRBC transfusion on the occurrence of clinically relevant postoperative complications for LLR in the PSS. Previous research by Tranchart et al., which also employed multivariate analysis to assess risk factors for postoperative complications after LLR, had fewer patients undergoing resection in the PSS. In their study, the association between intraoperative transfusion and clinically relevant postoperative complications was only observed in univariate analysis [35].

A potential strategy to minimize the intraoperative PRBC transfusion rate is preoperative screening for anemia. In cases of iron deficiency anemia, preoperative optimization with intravenous iron should be considered if time to surgery is limited or if the patient is intolerant or unresponsive to oral iron supplementation [36]. At the time of the analyses study period, this strategy was not routinely implemented at our center.

It is widely accepted that bleeding at the liver transection surface is influenced by the difference between intraabdominal and hepatic venous pressures, which in turn correlates with central venous pressure. Therefore, another strategy to reduce intraoperative PRBC transfusion is the implementation of the following intraoperative precautions, which are also recommended by current guidelines for managing bleeding in minimally invasive liver resection: (1) maintaining an intraabdominal pressure greater than 10 mmHg, (2) inflow control through portal triad clamping, and (3) outflow control by maintaining a low central venous pressure [37, 38]. Therefore we performed parenchymal transection under low central venous pressure (CVP) with the following conditions: (i) CO_2_ pneumoperitoneum maintained at 15 mmHg, (ii) a reversed Trendelenburg position, and (iii) intermittent Pringle maneuvers (with a maximum of 15 min of ischemia followed by 5 min of reperfusion) [22–26].

In previous studies on short-term outcomes after LLR for lesions in the PSS, the rate of clinically relevant complications (Clavien–Dindo ≥ III) varies from 2.3% [39] to 21.1% [40]. In line with the aforementioned literature, our study observed a rate of 18% (20 patients) with clinically relevant complications. The most frequent complication in our study was post-hepatectomy bile leakage, present in 10 patients (9%), which is consistent with previous data reporting PHBL rates between 3.6 and 6.8% [35, 41]. However, direct comparisons are challenging since most of the studies did not use the ISGLS classifications for specific complications. The median postoperative length of stay in our study was 6 days, consistent with previous reports on LLR of the PSS (range: 2 to 9 days) [42, 43].

Regarding the postoperative mortality, the rate in the literature ranged from 0% [9] up to 8% [44], with some authors reporting only 30-day mortality rates [42, 45]. In this study, the 30-day mortality rate was 4% (4 patients), while the overall 90-day mortality rate after LLR for lesions located in the PSS was 5% (six patients), and all except of one patient with iCC, suffered from HCC with liver cirrhosis. This is consistent with findings by Tranchart et al. [35], showing that HCC and liver cirrhosis were associated with most cases of postoperative mortality. Moreover, the primary cause of death in both studies was multiorgan failure.

Laparoscopic liver resection for lesions located in the PS segments is technically challenging and requires a standardized surgical approach. While various trocar placements are described in the current literature [43, 46–50], we established a totally laparoscopic standardized approach of 4 trocars, which are arranged along the reversed L-shape, which might be used for laparotomy [22–24, 26]. We use an anterior approach and the patient is placed in French position [51]. Compared to other studies, neither inter- or transcostal trocar placement nor a hand-assisted approaches (HAA) were performed in this study [43, 46, 47, 52]. Opening of the thoracic cavity might lead to a higher risk of complications and prolong operative time [48, 49]. However, the HAA is not required to be able to perform a safe LLR of PS segments and the possible advantage of the HAA (e.g., tactile sensation) might be overcome by the necessity of a lager incision bearing the risk of hand-port incisional hernia, increased postoperative pain, and air leakage [50].

There are some limitations in the present study. First, as a single-center study with a retrospective design, selection and reporting biases cannot be excluded. This may partly explain why well-known risk factors for Clavien–Dindo ≥ III complications, such as BMI and cirrhosis, were not identified as significant predictors in our analysis. The low overall incidence of these factors in our cohort may have contributed to this finding. Second, due to the short follow-up period, oncological long-term outcomes are missing. Third, larger sample sizes respectively metanalysis are needed to confirm our results. Fourth, due to the retrospective nature of the study, data on HbA1C levels, daily urinary sugar in diabetic patients, and malnutrition status are missing. Future research should address this gap. Fifth, future studies should explore targeted interventions to reduce failure-to-rescue rates due to sepsis. Strategies such as implementing a multidisciplinary team—including intensivists, nutritionists, and infectious disease specialists—could help optimize postoperative care, particularly for high-risk patients.

## Conclusion

Considering the postoperative outcomes of our study, in multivariate analyses ASA ≥ III, the presence of diabetes and intraoperative transfusion of packed red blood cells were risk factors associated with an increased risk of Clavien–Dindo grade ≥ III in patients undergoing LLR in PSS. Furthermore, avoidance of intraoperative transfusion of packed red blood cells should be a major objective of perioperative care of patients undergoing LLR in the PSS. Therefore, diabetes should be optimized as much as possible, and patients should be screened for anemia and provided with appropriate supplementation as part of preoperative planning for LLR in the PSS. In addition, patients with an ASA ≥ III should be distinctively regarded as high-risk patients and should be well informed about their increased risk of the occurrence of postoperative complications.

## Data Availability

Data underlying this study will be shared upon reasonable request to the corresponding author.
